# Capturing data for emergency department performance monitoring purposes

**DOI:** 10.12688/hrbopenres.12912.1

**Published:** 2019-08-13

**Authors:** Aileen McCabe, Maria Brenner, Philip Larkin, Sinéad Nic An Fhailí, Brenda Gannon, Ronan O'Sullivan, Abel Wakai

**Affiliations:** 1 National Children's Research Centre, Gate 5, Our Lady's Children's Hospital, Crumlin, Dublin 12, Ireland; 2 Emergency Care Research Unit (ECRU), HRB Centre for Primary Care Research, Mercer Building, Mercer Street Lower, Dublin 2, Ireland; 3 Department of Emergency Medicine, Tallaght University Hospital, Dublin 22, Ireland; 4 School of Nursing, Midwifery and Health Systems, Health Sciences Centre, University College Dublin, Dublin, Ireland; 5 School of Nursing and Midwifery, Trinity College Dublin, 24 D'Olier St. Dublin 2, Ireland; 6 Palliative and Supportive Care Service, Lausanne University Hospital, Lausanne, Switzerland; 7 Clinical Development and Analytics, Novartis Ireland, Dublin, Ireland; 8 Centre for Business and Economics of Health, University of Queensland, Brisbane, Australia; 9 Manchester Centre for Health Economics (MCHE), Institute of Population Health,, The University of Manchester, Manchester, UK; 10 Paediatric Emergency Research Unit (PERU), National Children’s Research Centre, Dublin 12, Ireland; 11 Bon Secours Hospital, Cork, Ireland; 12 Department of Emergency Medicine, Beaumont Hospital, Dublin 9, Ireland

**Keywords:** Emergency department, data collection; key performance indicators

## Abstract

**Background: **Good-quality data is required for valid and reliable key performance indicators. Little is known of the facilitators and barriers of capturing the required data for emergency department key performance indicators. This study aimed to explore and understand how current emergency department data collection systems relevant to emergency department key performance indicators are integrated into routine service delivery, and to identify the resources required to capture these data elements.

**Methods: **Following pilot testing, we conducted two focus groups with a multi-disciplinary panel of 14 emergency department stakeholders drawn from urban and rural emergency departments, respectively. Focus groups were analyzed using Attride–Stirling’s framework for thematic network analysis.

**Results:** The global theme “Understanding facilitators and barriers for emergency department data collection systems” emerged from three organizing themes: “understanding current emergency department data collection systems”; “achieving the ideal emergency department data capture system for the implementation of emergency department key performance indicators”; and “emergency department data capture systems for performance monitoring purposes within the wider context”.

**Conclusion: **The pathways to improving emergency department data capture systems for emergency department key performance indicators include upgrading emergency department information systems and investment in hardware technology and data managers. Educating stakeholders outside the emergency department regarding the importance of emergency department key performance indicators as hospital-wide performance indicators underpins the successful implementation of valid and reliable emergency department key performance indicators.

## Introduction

Key performance indicators (KPIs) identify where performance is good, where it meets desired standards, and where it requires improvement. Both the World Health Organization and, in Ireland, the Irish Health Information and Quality Authority (HIQA), recommend performing a feasibility analysis before using KPIs for healthcare services performance monitoring ^1, 2^. This is because collecting the relevant minimum data set (MDS) is always a limiting factor. MDS is defined as minimum core data identified to measure performance using a KPI ^2^. There is currently a knowledge gap regarding the facilitators and barriers of collecting MDS elements required to operationalize emergency department (ED) KPIs ^3^.

Using a qualitative focus group methodology, this study sought to explore and understand how current ED data collection systems for MDS elements relevant to ED KPIs were integrated into routine service delivery, and to identify the resources required to capture these elements.

## Methods

As part of a larger feasibility analysis project aimed at measuring the availability of the MDS elements relevant to 11 KPIs across 12 of the 29 public EDs in the Republic of Ireland ^4, 5^, two focus group interviews were conducted in August and November 2013. The focus groups involved EDs with predominantly urban and rural catchment areas.

### Ethical approval

The study was approved by the research ethics committees in Our Lady’s Children’s Hospital, Crumlin, Dublin and University College Cork (UCC).

### Participant selection and recruitment

Purposive sampling was used to identify and recruit key stakeholders involved in the process of collecting data elements relevant to ED KPIs. To enhance the study’s external validity, staff from EDs ( Table 1) with predominantly urban or rural catchment areas were selected for participation.

**Table 1.** Focus group participants.

Focus Group A (Urban EDs)	Focus Group B (Rural EDs)
Consultant in emergency medicine	Consultant in emergency medicine
Consultant in emergency medicine	Consultant in emergency medicine
Clinical Nurse Manager II	Consultant in emergency medicine
ED Data Manager	Consultant in emergency medicine
ED Business Manager	Senior ED Administrator
ED Clinical Nurse Manager III	ED Clinical Nurse Manager II
ED Receptionist	ED Staff Nurse

ED, emergency department.

A letter of invitation and a participant information sheet was sent to potential focus group participants to enable an informed choice regarding participation ^6^. Participation was entirely voluntary, although the participants were provided with refreshments and their travel expenses were reimbursed.

### Pilot testing

An interview topic guide (available as *Extended data* ^7^) was developed by an expert panel drawn from emergency medicine (EM), health services research (HSR) and health economics ^8^. The topic guide enabled structure and sequence to the questions posed, while at the same time, offering scope for development, clarification and exploration ^9^.

A pilot focus group interview was successfully conducted with ED staff and managers to test and refine the robustness of the topic guide, and to practice and enhance the quality of facilitation in terms of questioning, probing and guiding the discussion.

### Consent

Prior to each focus group, written informed consent was taken from participants.

### Data collection

The urban and rural focus groups were conducted in private rooms within a university (Dublin) and a hotel (Athlone), respectively. P.L. (PhD) moderated the urban focus group, while A.M. (MB BCH BAO, MRCEM, MSc) moderated the rural focus group. Three observers were present for both focus groups: a health economist research fellow, the project manager (S.N.A.F.) and a research assistant. Participants were briefed on the purpose of the study prior to the commencement of the focus groups. The majority of participants were familiar with authors A.M. and A.W. owing to their roles as EM physicians.

Digital audiotapes were used to record the focus group discussions. Topics were introduced with broad general statements or questions and the group was asked to discuss each issue with reference to their own experiences, decisions and practices, whilst reflecting on those of the other group members. Probing, clarifying and interpreting questions were used when necessary. Integral to the method, the facilitators were mindful of potential group dynamics, such as dominance of individuals and silent participants ^10^.

The moderators of the focus groups made short reflective notes following the interview to record observations of factors like group dynamics, group mood, tone of the discussion and key points to emerge in the discussion.

Sharing experiences in a mutual and supportive environment generated rich, critical information on the topic ^11^. Participant feedback and team reflection confirmed that the process enabled interactive dialogue within the focus groups with the emergence of congruent themes on issues pertaining to collecting the data required for operationalizing ED KPIs. Neither focus group interview ended until data saturation was achieved.

### Data analysis

Focus groups data were analyzed using Attride-Stirling’s thematic approach ^12^. This approach systematizes the extraction of basic themes (lowest-order premises evident in the text), organizing themes (categories of basic themes grouped together to summarize more abstract principles) and global themes (super-ordinate themes encapsulating the principal metaphors in the text as a whole). These are then represented as web-like maps depicting the salient themes at each of the three levels, and illustrating the relationships between them. One data coder was responsible for coding the data; data were managed manually without software.

Qualitative measures of rigor (credibility, authenticity, accuracy, confirmability and transferability) were applied to the data ^13, 14^. Seeking contradictory evidence and diverse experiences is essential to achieving a complete or exhaustive exploration of a phenomenon ^15^. The use of purposive sampling addressed the need to include both experienced voices and contradictory evidence. Credibility of the data was established through prolonged engagement with the topic and inclusion of expert respondents who had experience of capturing and mining ED data ^12^. With regards to authenticity, participants engaged based on their unique experiences, shared in relation to those of others and evidenced in multiple realities contained in the data collected. Data accuracy was established through creating verbatim transcripts of the digital audio files, which were returned to the study team for review ^9, 16^.

## Results

### Characteristics of focus group participants

The urban focus group lasted 91 and the rural focus group lasted 109 minutes. In total, seven respondents attended the urban ED focus group and the rural ED focus group interviews, respectively. One health economist and one consultant in EM participated in both focus groups, as did three observers; a health economist research fellow, the project manager and a research assistant. All other respondents (N=14) participated in one focus group only as presented in Table 1.

### Thematic network analysis

**Figure 1.**
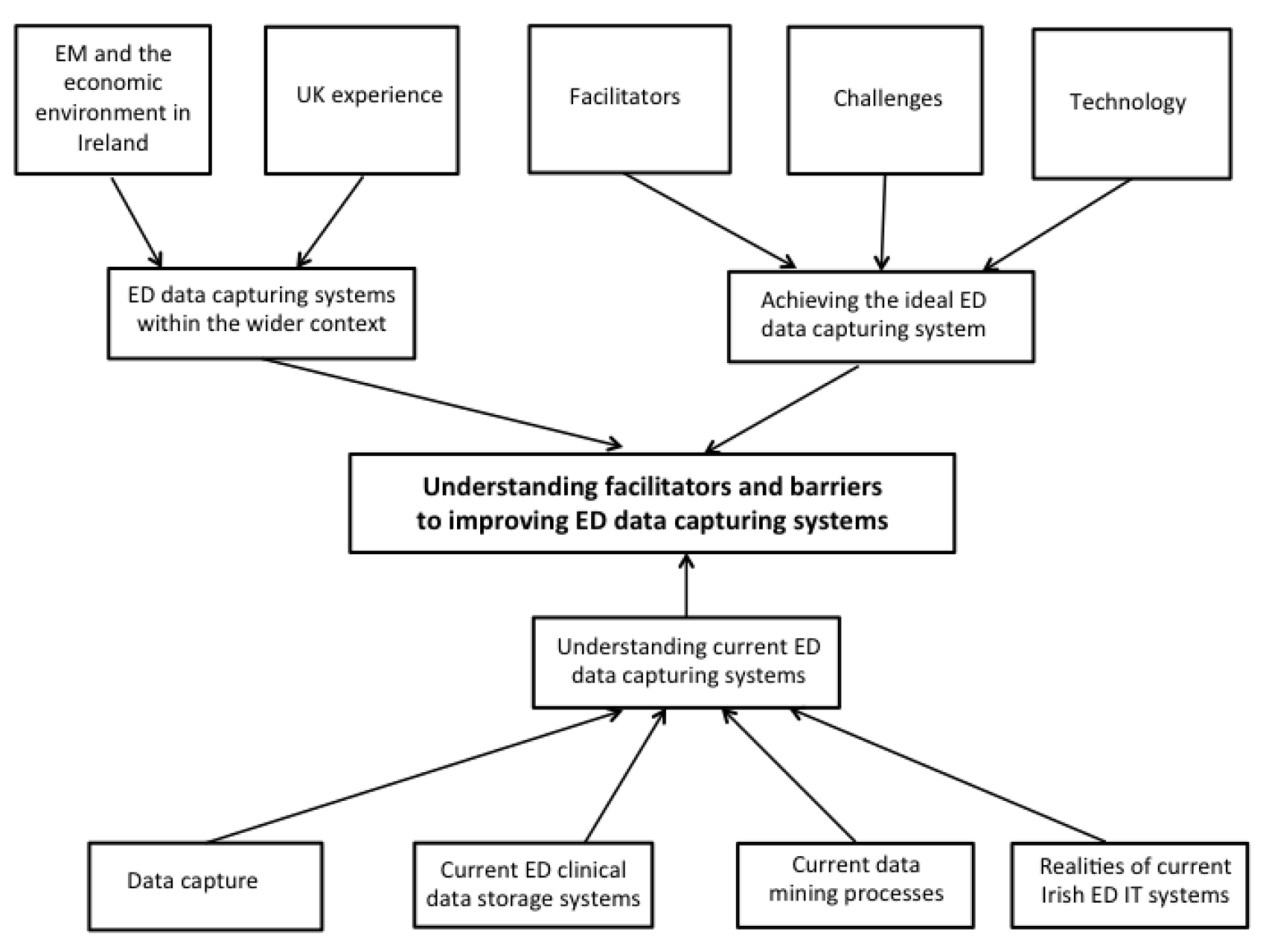
Understanding facilitators and barriers for emergency department (ED) data capture systems. EM, emergency medicine.

The global theme “understanding facilitators and barriers for ED data capture systems” emerged from three organizing themes as follows ( Figure 1).

### Understanding current ED data capture systems

This theme reflected current ED data collection, storage and mining practices and encompassed the basic themes of ED data capture, data storage, data mining processes and information technology support. Participants described patient clinical data capture in the ED as a combination of manual and electronic methods. In the public hospitals (run by the Irish service provider, the Health Service Executive [HSE] ^3^), EDs used iSoft Integrated Patient Management System (IPMS®) to capture and collate demographic data.

In others, bespoke ED Information Systems (EDIS) such as Symphony® (EMIS Health, UK) or MAXIMS® (IMS MAXIMS, United Kingdom) were used. In general, on patient arrival, administrative staff record patient demographic details on the EDIS. Thereafter, electronic data capture varied between the participant hospitals depending on the EDIS being employed. For example, laboratory and radiology orders were generally captured electronically but handwritten ED clinical records captured ED clinical assessment:

“A lot of data captured within the department is still manual.” (Urban 5)

Participants recognized the importance of routinely collecting reliable data to accurately reflect ED performance:

“If you use the electronic patient tracking system and whatever element of your patient record that is electronic… the data you will get will be real time and it will be accurate.” (Urban 3)

Conversely, one participant cautioned regarding the capture of invalid data:

“It is captured but it is meaningless. It is worse than not being captured and that goes for a lot of places.” (Urban 3)

There was a general consensus that electronic data capture, although challenging, minimized human error and hence, is more reliable than manual data capture:

“So that would give you the proper discharge time instead of relying on somebody to write it on a card… You need to force people to capture it at the source electronically.” (Urban 6)

Wide variance in data storage methods was reported. ED clinical data is either stored as hard copies or scanned and stored digitally. Participants highlighted several issues associated with this practice, including missing records, limited on-site storage facilities and the cost of outsourcing data storage space.

It was further noted that whilst an electronic patient record (EPR) system was ideal, scanning and digital storage was seen as superior to manual storage of clinical records. Where it was practiced, ED clinical records were generally scanned in an unstructured fashion.

For this study, the term ‘data mining’ was used to refer to the extraction of information from a database for the purpose of implementing ED KPIs. A number of efficient data mining software packages using predefined reports were identified in the focus group interviews. However, only a minority of staff, if any, were skilled in their use. Low prioritization given to reporting for EM at a hospital level was also reported:

“…the problem is that there are so many demands on these people, pulling them in all kinds of directions so evolution of the reporting for emergency medicine is low on their priority list.” (Rural 4)

At the time of the study there was only one dedicated ED data manager in Ireland, a significant barrier to data management for the implementation of valid and reliable ED KPIs. Reliance on staff from other hospital departments such as information technology (IT) or the statistics department to perform data mining was also noted:

“The other barrier we have is not having a dedicated data manager so you are relying on people who know how to do it; to do it almost as a favour.” (Urban 3)

Participants reported that EDIS offered limited functionality and downloading large data files was cumbersome and time-consuming. Databases such as IPMS®, designed for storing demographic data, provided visually useful if limited data for patient flow management or work flow planning.

Many alluded to local challenges regarding the IT system including upgrade delays, insufficient workstations and limited space to install further computers.

Where the HSE’s national iSoft IPMS® system is used, there was little technical support to troubleshoot problems at a local ED level. Central faults or maintenance was very disruptive:

“But yes, bad and all as our old legacy system was, and it was about to die but at least we had someone on-site… Whereas now we’ve heard it’s a national problem more times than you’d want to remember.” (Rural 4)

The national Emergency Medicine Programme (EMP) is the most comprehensive and ambitious strategic plan for emergency care ever undertaken in Ireland ^3^. It’s aim is to improve the safety and quality of care and reduce ED waiting times for patients throughout the country. The EMP has proposed a bespoke national EDIS system ^3^, rollout has been stalled. Of note, the national HSE’s IPMS had no input from EM into its implementation. There was also scant engagement between the designers of the system and the end-users, and training was rolled out in a cascade fashion:

“So there have been no sessions from the people who designed the system, IPMS, to come and teach the people who are using the system at the front-end how to actually use it. It’ll all be left to the people to cascade down how to use the system.” (Rural 5)

### ED data collection systems within the wider context

The second organizing theme reflected the economic and political factors impacting on ED data collection. Participants noted the challenging and unpredictable working conditions for ED staff work in and the consequences for ED data collection systems:

“It is about having the time actually to do everything. If two people are falling off a trolley and you are trying to click something on a computer, which are you going to pick?” (Urban 5)

ED overcrowding was referenced in both focus groups as a common challenge facing ED data collection:

“I think if you have a very, very busy department and you have a lot of people to look after …I think sometimes the data capture can be a lot more difficult than it would be.” (Urban 5)

Engagement by other key hospital stakeholders with ED performance measures as hospital performance measures was highlighted in the rural focus group:

“And I think there’s a bigger issue...It is the necessity for the hospital to buy into the fact that this is a hospital performance measurement. It’s not an emergency department measurement.” ** (Rural 3)

Participants drew comparisons with the UK National Health Service (NHS) in improving performance monitoring systems. One EM consultant who recently worked in the NHS, described the cultural difference between the Irish and British health care systems in their approach to performance monitoring:

“I don’t think there has been that much of a culture of looking at the notes for performance and others whereas I come from the NHS which is all about numbers and performance.” (Rural 5)

Significant monetary investment was evident in NHS performance monitoring initiatives in recent years to achieve mandated targets. Financial penalties imposed on hospitals for not meeting targets was a significant success factor. This was contrasted with the Irish system where no such penalties exist.

Having a cohort of performance managers to drive ED performance management within the NHS was critical:

“We would have performance managers who would go round and generate the reports, bring them to every single meeting, they’d be able to say – you need to focus here, you need to do this, you need to do that.” (Rural 5)

### Achieving the ideal ED data collection system

The third organizing theme focused on the areas of facilitators, barriers and health IT (HIT).

The lack of substantial upfront investment was noted as being a significant barrier to improving ED data collection systems necessary for the implementation of valid and reliable KPIs. With a poor culture of information and communications technology (ICT) spending within the health service, one participant advocated costing current waste as a tool to justify investment in ED data collection systems.

“I’d love to see it like that waste being costed because I think you would be able to prove relatively easily that an upfront investment…” (Rural 4)

Lack of implementation of standardized and agreed definitions of all ED operations is a significant barrier going forward in terms of ED data collection systems across Ireland. One suggested solution was to utilize the EMP definitions ^3^:

“It’s because people can count things to suit themselves. There needs to be clear definitions of what a new patient is, what a return, unscheduled or a review patient is… they are defined in the EMP programme document.” (Rural 4)

Engagement by key hospital stakeholders with ED performance measures as hospital performance measures was a recurrent theme. This engagement would improve data capture by other hospital staff on an EDIS and drive improvement in outcome measures relevant to patients admitted to hospital wards from the ED:

“You know, everybody in the hospital sees the 6- and 9-hour target, it’s all you guys in the ED and you’re only doing this so that you can meet your target in the ED. I don’t think that people actually realise that this is a national… it’s a hospital target, it’s not an ED thing, it’s a hospital measure.” (Rural 5)

The potential for ICT to improve ED data collection systems and so minimize human error was highlighted. Suggested solutions included radio-frequency identification (RFID) technology, barcoding and global positioning system (GPS) technology to record patient and staff movement. Moving to complete electronic data capture and particularly an electronic health record (EHR) system with fully integrated clinical, laboratory, radiology and ED triage data was considered desirable.

Currently used software systems were not considered user-friendly:

“I think when you have a system that is as clunky, as unattractive and unintuitive as most healthcare systems are, people have no interest in really using it, they don’t see any relevance to it and it’s also too complicated, it takes too long to go through every step.” (Rural 5)

## Discussion

The aim of this qualitative study was to explore and describe how well current ED data capturing systems for MDS relevant to ED KPIs are integrated into routine service delivery and to identify the required resources. This study revealed that current Irish ED data capturing systems are complex and heterogeneous with multiple facilitators and barriers identified.

This study confirmed that the required ED data collection systems for the relevant MDS elements are not currently well integrated into routine service delivery. Indeed, the participants highlighted a number of infrastructural limitations, the most notable of which was that the currently available information systems in many EDs had a limited capacity to capture clinical data and ED activity. This finding was consistent with the results of a Delphi consensus study of Irish EM consultants that found that the presence of a dedicated EDIS was the highest-rated ED KPI ^17^.

The study also highlighted a lack of dedicated data managers in Irish EDs, a vital facilitator to drive improvement in ED data collection systems. To the best of our knowledge, there is no published literature demonstrating the benefits and cost-effectiveness of a dedicated ED data manager regarding the implementation of ED KPIs. However, it is intuitively logical that an ED data manager will facilitate more efficient capture of MDS elements directly relevant to ED KPIs.

Ireland’s experience of a lack of a national comprehensive approach to ED data collection systems and implementation of EHR systems appears to be replicated in a systematic review by Boonstra *et al*. ^18^. The introduction of an EHR system was identified as a facilitator for capturing the relevant MDS elements in the ED. However, there is evidence that introduction of an EHR system can be associated with a deterioration in meeting standards outlined in ED KPIs ^19– 21^. Initial adjustment to an EHR system may increase documentation time but as staff become more familiar with the system, it may ultimately improve work flow ^22^.

The finding in this study that the lack of hospital and health service organisational ownership of ED KPIs contributes to negative effects on staff wellbeing and patient safety is consistent with previous studies ^23, 24^. The need for strong management, leadership and cultural change were considered important facilitators. Furthermore, the importance of implementing agreed ED operational definitions was identified as being important. This finding is consistent with previous studies which have also highlighted the need to standardize language and terminology as a means of standardizing ED performance monitoring ^25^.

### Strengths

To the best of our knowledge, this is the first study exploring and describing the experience of capturing relevant MDS elements relevant to ED KPIs from a multi-disciplinary frontline ED staff perspective. Qualitative research methodologies such as focus groups are ideal to identify facilitators and barriers for processes in the highly specialized, complex and unique ED team-oriented patient care environment that may not readily be amenable through quantitative approaches ^26– 28^. Focus groups provide major insights into attitudes, beliefs and opinions, are relatively inexpensive and generate a rich dataset as participants build on each other’s ideas ^26, 27, 29, 30^.

### Limitations

Although, purposive sampling was considered useful in identifying and recruiting healthcare professionals into the focus group, not all of the key stakeholders were represented. For example, neither HSE management nor hospital medical record department staff was able to attend due to lack of availability and it is possible that the focus groups did not capture representative views for these relevant stakeholders. However, the participants presented a range of views and neither focus group interview ended until data saturation (i.e. no new data was captured).

## Conclusions

This study identified ICT investment, employing more ED data managers and engaging stakeholders outside the ED as important facilitators for the capture of the MDS elements that underpin valid and reliable ED KPIs.

## Data availability

### Underlying data

Open Science Framework: Capturing data for emergency department performance monitoring purposes. https://doi.org/10.17605/OSF.IO/93K7A ^7^.

This project contains the following underlying data:

- Rural focus group transcript .pdf
- Urban focus group transcript .pdf

### Extended data

Open Science Framework: Capturing data for emergency department performance monitoring purposes. https://doi.org/10.17605/OSF.IO/93K7A ^7^.

This contains the following extended data:

- Focus Group Interview Guide.docx

Data are available under the terms of the Creative Commons Zero “No rights reserved” data waiver (CC0 1.0 Public domain dedication).

## Author information

Further clarifications of the authors’ previous and present affiliations if they have changed since the study was conducted are listed below:

**McCabe A**: National Children’s Research Centre, Gate 5, Our Lady’s Children’s Hospital, Crumlin, Dublin 12, Ireland.

Emergency Care Research Unit (ECRU), HRB Centre for Primary Care Research, Mercer Building, Mercer Street Lower, Dublin 2, Ireland.

*Present address*: Department of Emergency Medicine, Tallaght University Hospital, Tallaght, Dublin 22, Ireland.

**Brenner M**: School of Nursing, Midwifery and Health Systems, Health Sciences Centre, University College Dublin, Ireland.

*Present address*: School of Nursing and Midwifery, Trinity College Dublin, 24 D’Olier St. Dublin 2, Ireland.

**Larkin P**: School of Nursing, Midwifery and Health Systems, Health Sciences Centre, University College Dublin, Ireland.

*Present address*: Palliative and Supportive Care Service, Lausanne University Hospital, Switzerland.

**Nic An Fhailí S**: National Children’s Research Centre, Gate 5, Our Lady’s Children’s Hospital, Crumlin, Dublin 12, Ireland.

*Present address*: Clinical Development and Analytics, Novartis Ireland, Dublin, Ireland.

**Gannon B**: Manchester Centre for Health Economics (MCHE), Institute of Population Health, The University of Manchester, United Kingdom.

*Present address*: Centre for Business and Economics of Health, University of Queensland, Australia.
