# Supplementary material for: Capturing data for emergency department performance monitoring purposes
Source: HRB Open Res. 2019 Aug 13;2:18. [Version 1] doi: 10.12688/hrbopenres.12912.1 (PMC7490568; doi:10.12688/hrbopenres.12912.1)
Supplement: Peer review history

# Peer review: Reviewer response for version 1

**DOI:** 10.21956/hrbopenres.13990.r27864

**Published:** 2020-09-14

**Authors:** Raj M Ratwani

**Affiliations:** 1 School of Medicine, Georgetown University, Washington, DC, USA; 2 MedStar Health National Center for Human Factors in Healthcare, Washington, DC, USA

The authors present a study investigating how current emergency department data collection systems serve to fill the measurement of key performance indicators (KPIs), with a focus on integration of these systems with routine service delivery. A qualitative analysis of subject matter expert focus groups was conducted. The authors identified several facilitators and barriers that are organized around different themes.

 Overall, the manuscript is well written. The method is generally sound, and the results are interesting. A few things to consider:

 Methods

- What constituted reaching data saturation?
- From my experience, most qualitative studies have 2 coders that reach inter-rater reliability. Why was only one coder used?

Results

- Figure 1 - would be good to indicate the basic themes, organizing themes, etc. I was little bit confused by what each box was actually representing.
- Some of the labels for the organizing themes are confusing, especially when just reading the abstract (results). Once I read the entire paper the labels made more sense. This is a minor point, but you may want to reexamine the terminology.

Is the work clearly and accurately presented and does it cite the current literature?

Yes

If applicable, is the statistical analysis and its interpretation appropriate?

Yes

Are all the source data underlying the results available to ensure full reproducibility?

Yes

Is the study design appropriate and is the work technically sound?

Yes

Are the conclusions drawn adequately supported by the results?

Yes

Are sufficient details of methods and analysis provided to allow replication by others?

Partly

Reviewer Expertise:

Health information technology, patient safety, usability, human factors.

I confirm that I have read this submission and believe that I have an appropriate level of expertise to confirm that it is of an acceptable scientific standard.

# Peer review: Reviewer response for version 1

**DOI:** 10.21956/hrbopenres.13990.r27355

**Published:** 2020-05-12

**Authors:** Arkalgud Ramaprasad

**Affiliations:** 1 College of Business Administration, University of Illinois at Chicago, Chicago, IL, USA; 2 Ramaiah Public Policy Center, Bengaluru, India

The study belongs to the ‘grounded research’ genre using focus group discussions. It is well defined and conducted. The extraction and interpretation of the themes are well aligned with the procedures of the genre. While the conclusions drawn are supported by the results, they are limited by the grounded framing of the problem. It is inherent in the genre of research and not particular to this paper. However, the authors may want to add a qualifying note about the limitation. I shall explain the issue with reference to the ontology here.

 The problem the authors are addressing may be specified fully as: Information System Impetus to Capture Data for Emergency Department (ED) Key Performance Indicators (KPI). The ontology articulates this problem in structured natural English as follows.

 The data on ED KPIs must be reliable, valid, (of high) quality, timely, and comprehensive. These attributes are mentioned in the paper and shown in the rightmost column of the ontology. Capturing the data with the above attributes entails the traditional functions of an information system: acquire, store, retrieve, process, distribute, and delete. Many of these or their synonyms are mentioned in the focus group discussions. The paper is focused on the barriers and facilitators to capture the data. They are labeled impetus in the ontology, with an additional neutral middle labeled the norm – neither a barrier nor a driver. The information system (IS) itself is characterized by its structural elements – architecture, infrastructure, network, system, service, policy, process, and personnel – listed in the first column. This taxonomy is based on the common body of knowledge in information systems. Some of these elements (or their synonyms) are mentioned in the focus group data quoted – but not all.

 The concatenation of an element from each column together with the adjacent phrases/words forms a natural English sentence that describes an information system impetus pathway to capture data for ED KPI. There are 720 pathways encapsulated in the ontology – they are all the potential drivers, norms, and barriers to capture data for ED KPI.

 Without a logical framework such as the ontology framing the research, the findings are subject to significant errors of omission. For example, there may be IS policy barriers to distribute timely data for ED KPI. Without the ontology it would not be possible to surface data about it in the focus group discussion. And, its absence may not be noticed. Latently, that may be a critical issue – although absent in the discussion. There may be many other similar latent critical issues that need to be addressed to solve the problem.

 One could systematically map the focus group discussions onto an ontology such as the one presented to discover the pathways emphasized frequently, infrequently, and never. The data the authors have is rich – they may discover themes and sub-themes in addition to the ones mentioned in the paper. The mapping may become a study by itself. However, for the present paper, the authors may want to extend their section on the limitations to highlight the above limitation of the method and hence the findings. And, suggest how the limitation may be overcome in the future.

Is the work clearly and accurately presented and does it cite the current literature?

Yes

If applicable, is the statistical analysis and its interpretation appropriate?

Not applicable

Are all the source data underlying the results available to ensure full reproducibility?

Yes

Is the study design appropriate and is the work technically sound?

Yes

Are the conclusions drawn adequately supported by the results?

Yes

Are sufficient details of methods and analysis provided to allow replication by others?

Yes

Reviewer Expertise:

Information systems, ontological meta analysis and synthesis.

I confirm that I have read this submission and believe that I have an appropriate level of expertise to confirm that it is of an acceptable scientific standard.
